# Photonic scheme of discrete quantum Fourier transform for quantum algorithms via quantum dots

**DOI:** 10.1038/s41598-019-48695-z

**Published:** 2019-08-27

**Authors:** Jino Heo, Kitak Won, Hyung-Jin Yang, Jong-Phil Hong, Seong-Gon Choi

**Affiliations:** 1 College of Electrical and Computer Engineering, Chungbuk National University, Chungdae-ro 1, Seowon-Gu, Cheongju Republic of Korea; 2 Program in Bio-medical Science, Korea University, Sejong, 30019 Republic of Korea; 3 Department of Natural Science, Republic of Korea Air-Force Academy, Cheongju, 28187 Republic of Korea; 4 Department of Physics, Korea University, Sejong, 339-700 Republic of Korea

**Keywords:** Quantum information, Quantum optics, Computational science

## Abstract

We propose an optical scheme of discrete quantum Fourier transform (DQFT) via ancillary systems using quantum dots (QDs) confined in single-sided cavities (QD-cavity systems). In our DQFT scheme, the main component is a controlled-rotation k (CRk) gate, which utilizes the interactions between photons and QDs, consisting of two QD-cavity systems. Since the proposed CRk gate can be experimentally implemented with high efficiency and reliable performance, the scalability of multi-qubit DQFT scheme can also be realized through the simple composition of the proposed CRk gates via the QD-cavity systems. Subsequently, in order to demonstrate the performance of the CRk gate, we analyze the interaction between a photon and a QD-cavity system, and then indicate the condition to be efficient CRk gate with feasibility under vacuum noise and sideband leakage.

## Introduction

In various quantum algorithms and quantum computations, such as quantum phase estimation algorithm^1–3^, the factoring problem^4–7^, the discrete logarithm problem^2,4,8,9^, and the hidden subgroup problem^10–12^, a discrete quantum Fourier transform (DQFT)^2,13–19^ plays a critical role in accomplishing quantum information processing. Thus, for the experimental implementation of DQFT, a variety of physical resources have been used, including those based on linear optical systems^20–22^, nonlinear optical systems^17,23–25^, nuclear magnetic resonance or ion trap systems^26–29^, superconducting circuits^30^, and cavity-QED^15,31–33^.

In addition, for quantum information processing schemes, many researchers have theoretically proposed and experimentally designed quantum controlled gates^34–43^, which can apply an arbitrary operation to a target qubit according to a control qubit, via nonlinearly optical resources. In addition, in the DQFT scheme, the reliable quantum controlled operations are crucial components for transforming an input state of qubit into the linearly combined states of qubit. In particular, preserving the coherence against the decoherence effect and the extension of coherence time in quantum state are the most important challenges for the reliable performance of quantum controlled operation with the reliable performance.

From this point of view, for the coherence of quantum system, optical systems of micropillar cavities have been widely used to construct quantum controlled gates^38–42^. In particular, quantum information in the quantum dot (QD)-cavity system, which consists of an excess electron and a negatively charged exciton (X^−^) confined within an optical cavity^40,42,44–66^, can be well isolated from the environment for a long electron-spin coherence time (${{\rm{T}}}_{{\rm{2}}}^{{\rm{e}}}$~μs)^57–62^ as well as a limited spin relaxation period (${{\rm{T}}}_{{\rm{1}}}^{{\rm{e}}}$~ms)^63–66^. Therefore, quantum controlled gates^38,39,41,48,51,67–69^ have been proposed via the QD-cavity system between photon-photon, electron-electron, and electron-photon.

In this paper, we design an optical controlled-rotation k (CRk) gate based on the interactions between two photons and two QD-cavity systems, as well as linearly optical devices. The proposed CRk gate can be directly applicable to comprise a scheme of DQFT for quantum computation and quantum algorithm. According to the expansion to arrange the CRk gates and the alternation of rotation operators (k), we can achieve scalability of the DQFT scheme, because our CRk gate using the QD-cavity systems serves as the basic module of the multi-qubit DQFT scheme. Subsequently, for the deterministic DQFT scheme, we analyze the efficiency and performance of the CRk gate using the interaction of photon-electron in QD under vacuum noise in the QD-dipole operation, and leaky modes (sideband leakage and absorption)^40,42,53–56^. Consequently, our DQFT scheme via the CRk gates shows scalability and experimental feasibility in practice.

## Theoretical Circuit of Discrete Quantum Fourier Transform

The arbitrary quantum state, |*ϕ*〉, can be transformed by the operation of DQFT^2,13–19^, as follows:

1 $$\begin{array}{ccc}|\varphi \rangle =\mathop{\sum }\limits_{m=0}^{{2}^{t}-1}{\alpha }_{m}|m\rangle  & \mathop{\to }\limits^{{\rm{DQFT}}} & \mathop{\sum }\limits_{f=0}^{{2}^{t}-1}{\beta }_{f}|f\rangle \end{array},$$

where ${\beta }_{f}=[{\sum }_{m=0}^{{2}^{t}-1}{\alpha }_{m}{e}^{2\pi i(mf)/{2}^{t}}]/\sqrt{{2}^{t}}$. Thus, DQFT on an orthonormal basis, |0〉, |1〉, …, |2^*t*^ − 1〉 is defined as an operator, U_DQFT_, as follows:

2 $${{\rm{U}}}_{{\rm{DQFT}}}|m\rangle =\frac{1}{\sqrt{{2}^{t}}}\mathop{\sum }\limits_{f=0}^{{2}^{t}-1}{e}^{2\pi i(mf)/{2}^{t}}|f\rangle ,$$

where the |0〉, |1〉, …, |2^*t*^ − 1〉 is the orthonormal basis consisting of the quantum state on *t* qubits. Using binary representation, state |*m*〉 can be written as |*m*〉 ↔ |*j*_1_〉 ⊗ … ⊗ |*j*_*t*_〉 where *m* ≡ *j*_1_ × 2^*t*−1^ + *j*_2_ × 2^*t*−2^ + $\cdots$ + *j*_*t*_ × 2^0^ and *j*_*n*_ ∈ {0, 1}. Thus, the product representation of DQFT on qubits can be given by

3 $$|m\rangle \leftrightarrow |{j}_{1}\rangle \otimes \cdots \otimes |{j}_{t}\rangle \mathop{\Rightarrow }\limits^{{\rm{D}}{\rm{Q}}{\rm{F}}{\rm{T}}}\Rightarrow \,\frac{1}{\sqrt{{2}^{t}}}(|0{\rangle }_{1}+{e}^{2\pi i(0.{j}_{t})}|1{\rangle }_{1})\otimes (|0{\rangle }_{2}+{e}^{2\pi i(0.{j}_{t-1}{j}_{t})}|1{\rangle }_{2})\otimes \cdots \otimes (|0{\rangle }_{t}+{e}^{2\pi i(0.{j}_{1}{j}_{2}\cdots \cdot {j}_{t})}|1{\rangle }_{t}),$$

where the notation, 0.*j*_1_*j*_2_ $\cdots$ *j*_*t*−1_*j*_*t*_, is the binary fraction as 0.*j*_1_*j*_2_ $\cdots$ *j*_*t*_ ≡ *j*_1_ × 2^−1^ + *j*_2_ × 2^−2^ + $\cdots$ + *j*_*t*_ × 2^−*t*^.

**Figure 1:**
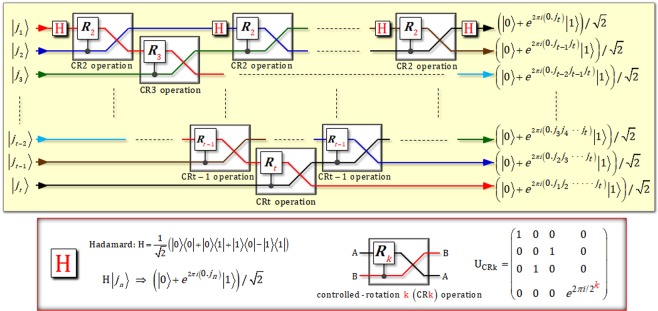
Plot presents the theoretical circuit to implement the operation of DQFT on *t* qubits. This circuit consists of controlled-rotation k [CRk (*k* = 2, …, *t*)] operations and Hadamard operations for DQFT on *t* qubits.

Figure 1 shows the quantum circuit of DQFT to transform the input state, $|{j}_{1}\rangle \otimes |{j}_{2}\rangle \otimes \cdots \otimes |{j}_{t}\rangle$, into the product representation of DQFT on *t* qubits. As shown in Fig. 1, the significant important components in a circuit of DQFT are the CRk operations, U_CRk_, between two qubits to transform the input state. For example, let us assume an initial state of two qubits as ${|\varphi \rangle }_{{\rm{in}}}={x}_{1}{|0\rangle }_{{\rm{A}}}{|0\rangle }_{{\rm{B}}}+{x}_{2}{|0\rangle }_{{\rm{A}}}{|1\rangle }_{{\rm{B}}}+{x}_{3}{|1\rangle }_{{\rm{A}}}{|0\rangle }_{{\rm{B}}}+{x}_{4}{|1\rangle }_{{\rm{A}}}{|1\rangle }_{{\rm{B}}}$. As described in Fig. 1, the CRk operation (U_CRk_) can transform the initial state, |*ϕ*〉_in_, into a final state, |*ϕ*〉_fin_, as follows:

4 $$\begin{array}{ccc}{|\varphi \rangle }_{{\rm{in}}} & \mathop{\Rightarrow }\limits^{{\rm{CRk}}({{\rm{U}}}_{{\rm{CRk}}})} & {|\varphi \rangle }_{{\rm{fin}}}={x}_{1}{|0\rangle }_{{\rm{B}}}{|0\rangle }_{{\rm{A}}}+{x}_{2}{|1\rangle }_{{\rm{B}}}{|0\rangle }_{{\rm{A}}}+{x}_{3}{|0\rangle }_{{\rm{B}}}{|1\rangle }_{{\rm{A}}}+{x}_{4}{e}^{2\pi i/{2}^{k}}{|1\rangle }_{{\rm{B}}}{|1\rangle }_{{\rm{A}}},\end{array}$$

where the paths of the two qubits are swapped and the operation of rotation (*R*_*k*_) is applied into the target qubit when a control qubit is in state |1〉 by CRk operation (two qubits controlled operation). Thus, if the CRk (*k* = 2, …, *t*) and Hadamard operations are arranged as shown in Fig. 1, we can obtain the theoretical circuit of multi-qubit DQFT with scalability. Consequently, for the high efficiency and reliable performance of DQFT based on CRk operations, the main issue is to design the experimentally implemented CRk operation in a feasibility manner.

## Quantum Dot Inside Optical Cavity and Controlled-Rotation k Gate for Discrete Quantum Fourier Transform

### Interaction of photon and QD-cavity system

**Figure 2:**
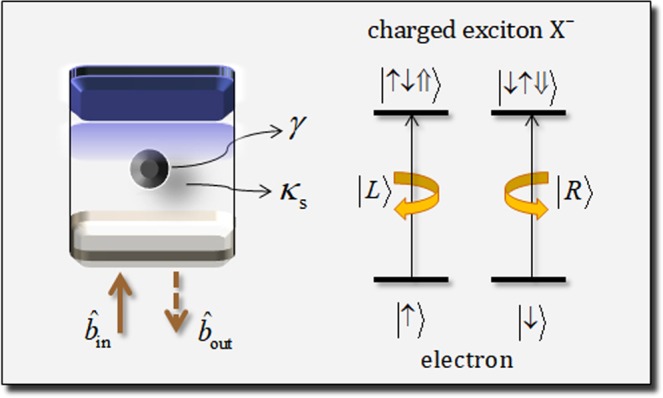
Singly charged QD within a single-sided cavity with a photon ${\hat{{b}}}_{{\rm{in}}}$ and ${\hat{{b}}}_{{\rm{out}}}$. Due to the spin selection rule, the interaction between a polarization of incident photon and a spin state of excess electron in QD can create the transition that the photon |*L*〉 drives the state as |↑〉 → |↑↓⇑〉 (or the photon |*R*〉 drives the state as |↓〉 → |↓↑⇓〉), where |↑〉 ≡ |+1/2〉, |↓〉 ≡ |−1/2〉 are the spin states of the excess electron while |⇑〉 and |⇓〉(*J*_*z*_ = +3/2, −3/2) represent heavy-hole spin states.

We introduce a QD-cavity system^42,44–52,63^, which can induce the interaction of a photon and a singly charged QD (a negatively charged exciton: X^−^) confined in a single-sided optical cavity, and a reflection operator ($\hat{{\rm{R}}}$) from the interaction between a photon and the QD-cavity system. Figure 2 represents that the QD-cavity system is composed of two GaAs/Al(Ga)As distributed Bragg reflections (DBRs: bottom DBR – partially reflective, and top DBR – 100% reflective) as a single-sided cavity. And QD is confined at the center of the single-sided cavity (between two DBRs), where *κ*_*s*_ and *γ* are the side-leakage rate of the cavity mode and the decay rate of X^−^ (two electrons bound to one hole), respectively. When a photon pulse is incident into the QD, an interaction occurs between the photon and an excess electron into the QD, according to the Pauli Exclusion Principle, where ${\hat{{b}}}_{{\rm{in}}}$ (${\hat{{b}}}_{{\rm{out}}}$) is the input (output) field operators of the photon pulse. As illustrated in Fig. 2, if the spin state of the excess electron in the QD is in the state |↑〉, then the polarization |*L*〉 of a photon can drive the state |↑↓⇑〉 of X^−^. Also, if the spin state |↓〉 of the excess electron in the QD and the polarization |*R*〉 of a photon, the interaction, the state |↓↑⇓〉, of X^−^ can be created. Let us assume the approximation of weak excitation with the ground state, 〈*σ*_*Z*_〉 = −1, in the QD for the steady state^42,45,46,50–52^. Then, due to the spin selection rule, we can calculate the reflection coefficient, *R*(*ω*), of the reflected photon and the QD from the Heisenberg equation of motion^70^, and also the reflectance, |*r*_h_| (|*r*_0_|), and phase shift *ϕ*_rh_ (*ϕ*_r0_) of the hot (cold) cavity, as follows:

5 $$R(\omega )=\frac{{\hat{{b}}}_{{\rm{out}}}}{{\hat{{b}}}_{{\rm{in}}}}=\frac{[i({\omega }_{{{\rm{X}}}^{-}}-\omega )+\gamma /2][i({\omega }_{c}-\omega )-\kappa /2+{\kappa }_{s}/2]+{g}^{2}}{[i({\omega }_{{{\rm{X}}}^{-}}-\omega )+\gamma /2][i({\omega }_{c}-\omega )+\kappa /2+{\kappa }_{s}/2]+{g}^{2}},$$

where ${\omega }_{{{\rm{X}}}^{-}}$, *ω*_*c*_, and *ω* are the frequencies of X^−^, cavity mode, and external field, respectively. Also, *g* is the coupling strength between X^−^ and cavity mode while *κ* is the decay rate of the cavity mode. In the interaction between a photon and an electron in the QD, when the spin state of the excess electron is in the state |↑〉(|↓〉), the polarization of the photon |*L*〉(|*R*〉) drives the hot cavity (the QD is coupled with the cavity: *g* ≠ 0), while the polarization of the photon |*R*〉(|*L*〉) feels the cold cavity (the QD is uncoupled with the cavity: *g* = 0). Thus, for the resonant interaction (${\omega }_{{{\rm{X}}}^{-}}={\omega }_{c}$), the reflection coefficients *R*_h_(*ω*) and *R*_0_(*ω*) can be calculated as follows:

6 $$\begin{array}{ccc}{\rm{h}}{\rm{o}}{\rm{t}}\,{\rm{c}}{\rm{a}}{\rm{v}}{\rm{i}}{\rm{t}}{\rm{y}}:{R}_{{\rm{h}}}(\omega )\equiv |{r}_{{\rm{h}}}(\omega )|\exp [i{\phi }_{{\rm{r}}{\rm{h}}}(\omega )] & = & \frac{[i({\omega }_{c}-\omega )+\gamma /2][i({\omega }_{c}-\omega )-\kappa /2+{\kappa }_{s}/2]+{g}^{2}}{[i({\omega }_{c}-\omega )+\gamma /2][i({\omega }_{c}-\omega )+\kappa /2+{\kappa }_{s}/2]+{g}^{2}}\\  & = & R(\omega ),\\ {\rm{c}}{\rm{o}}{\rm{l}}{\rm{d}}\,{\rm{c}}{\rm{a}}{\rm{v}}{\rm{i}}{\rm{t}}{\rm{y}}:{R}_{0}(\omega )\equiv |{r}_{0}(\omega )|\exp [i{\phi }_{{\rm{r}}0}(\omega )] & = & \frac{i({\omega }_{c}-\omega )-\kappa /2+{\kappa }_{s}/2}{i({\omega }_{c}-\omega )+\kappa /2+{\kappa }_{s}/2},\end{array}$$

where ${\phi }_{{\rm{r}}{\rm{h}}}\equiv \arg [{R}_{{\rm{h}}}]\,({\phi }_{{\rm{r}}0}\equiv \arg [{R}_{0}])$ is the phase shift in the hot (cold) cavity. Using these reflectances (|*r*_h_|, |*r*_0_|) and phase shifts (*ϕ*_rh_, *ϕ*_r0_) in the reflection coefficients (*R*_h_, *R*_0_), we can obtain the reflection operator, $\hat{{\rm{R}}}(\omega )$, with the resonant interaction (${\omega }_{{{\rm{X}}}^{-}}={\omega }_{c}$), as follows:

7 $$\hat{{\rm{R}}}(\omega )=|{r}_{{\rm{h}}}(\omega )|{e}^{i{\phi }_{{\rm{rh}}}(\omega )}(|R\rangle \langle R|\otimes |\downarrow \rangle \langle \downarrow |+|L\rangle \langle L|\otimes |\uparrow \rangle \langle \uparrow |)+|{r}_{{\rm{0}}}(\omega )|{e}^{i{\phi }_{{\rm{r0}}}(\omega )}(|R\rangle \langle R|\otimes |\uparrow \rangle \langle \uparrow |+|L\rangle \langle L|\otimes |\downarrow \rangle \langle \downarrow |).$$

When the experimental parameters are *κ*_*s*_ ≪ *κ* (small side-leakage rate) and *g* ≫ (*κ*, *γ*) (large coupling strength) with small *γ* (~several μ eV)^71–74^, two kinds of reflection operators (${\hat{{\rm{R}}}}^{1}$ and ${\hat{{\rm{R}}}}^{2}$), obtained by adjusting the frequencies of the external field, *ω*, and cavity mode, *ω*_*c*_, can be given as

8 $$\begin{array}{lll}\omega -{\omega }_{c}=\kappa /2 & : & {\hat{{\rm{R}}}}^{1}(\omega )\approx |R\rangle \langle R|\otimes |\downarrow \rangle \langle \downarrow |+|L\rangle \langle L|\otimes |\uparrow \rangle \langle \uparrow |-i|R\rangle \langle R|\otimes |\uparrow \rangle \langle \uparrow |-i|L\rangle \langle L|\otimes |\downarrow \rangle \langle \downarrow |,\\ \omega -{\omega }_{c}=0 & : & {\hat{{\rm{R}}}}^{2}(\omega )\approx |R\rangle \langle R|\otimes |\downarrow \rangle \langle \downarrow |+|L\rangle \langle L|\otimes |\uparrow \rangle \langle \uparrow |-|R\rangle \langle R|\otimes |\uparrow \rangle \langle \uparrow |-|L\rangle \langle L|\otimes |\downarrow \rangle \langle \downarrow |,\end{array}$$

where the reflectances and phase shifts are |*r*_h_| = |*r*_0_| ≈ 1(≈1) and *ϕ*_rh_ ≈ 0 (≈0), *ϕ*_r0_ ≈ −*π*/2 (≈*π*), respectively, in the detuning of frequency as *ω* − *ω*_*c*_ = *κ*/2 (*ω* − *ω*_*c*_ = 0) for *g*/*κ* = 2.4 and *γ*/*κ* = 0.1 with *κ*_*s*_ → 0 ^42,44–52,63^. Subsequently, we will utilize the interaction of the QD-cavity system, the reflection operators (${\hat{{\rm{R}}}}^{1}$ and ${\hat{{\rm{R}}}}^{2}$) in Eq. 8, on the CRk gate using the QD-cavity systems for the DQFT scheme.

### Design of CRk gate using two QD-cavity systems for DQFT scheme

In this section, we propose a feasible controlled-rotation k (CRk) gate using the interaction, in Sec. 3.1, of the QD-cavity system, in order to implement the CRk operation of Fig. 1 for direct applications in the DQFT scheme. First, we assume the definition of binary representation in the polarization of the photon as {|*R*〉, |*L*〉} ≡ {|0〉, |1〉}, and the relation between the circular- (|*R*〉 is right and |*L*〉 is left) and linear-polarization (|*H*〉 is horizontal and |*V*〉 is vertical), as $|R\rangle \equiv (|H\rangle +|V\rangle )/\sqrt{2}$ and $|L\rangle \equiv (|H\rangle -|V\rangle )/\sqrt{2}$.

**Figure 3:**
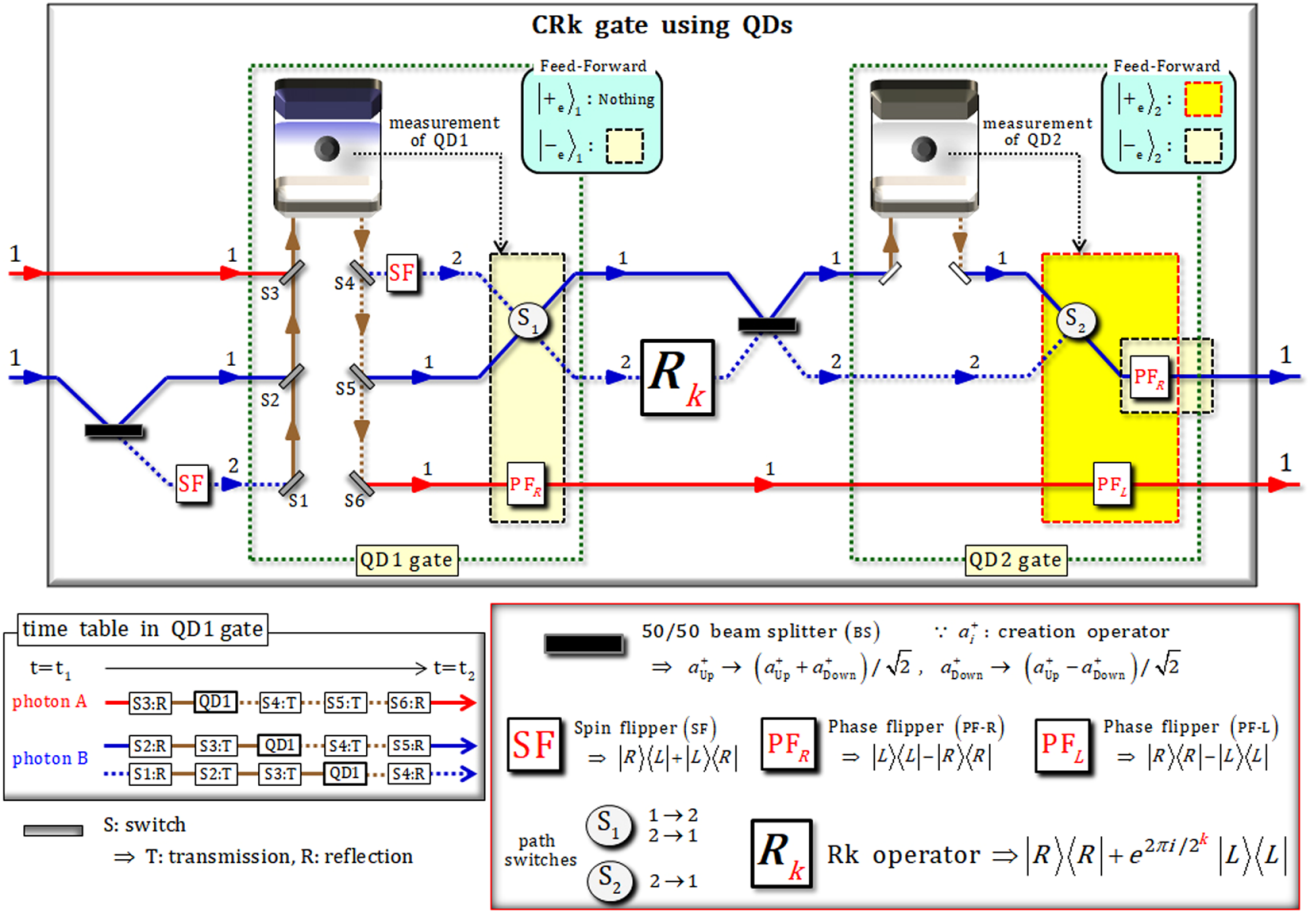
Controlled-rotation k (CRk) gate: For the implementation of the CRk operation illustrated in Fig. 1, this CRk gate employs two QD-cavity systems (QD1 and QD2 gates) and an Rk operator with linearly optical devices (BSs, SFs, and feed-forwards: PF-R, PF-L, path switches). In the QD1 gate, the interactions between photons (A and B) and QD1 are used as the reflection operator of ${\hat{{\rm{R}}}}^{1}$ (*ω* − *ω*_*c*_ = *κ*/2), in Eq. 8. Further, the operations of the switches (S1~S6) on the paths of photon A and B comply with the time table. After measuring QD1 (electron spin 1), the feed-forward (PF-R and path switch 1) is performed by the outcome of QD1. In the QD2 gate, the reflection operator of ${\hat{{\rm{R}}}}^{2}$ (*ω*−*ω*_*c*_ = 0), shown in Eq. 8, applies to the interaction of a photon B and QD2. Then, according to the outcome of measurement on QD2 (electron spin 2), PF-R, PF-L, and path switch 2 (feed-forward) are performed on photon A and B.

Figure 3 shows a schematic of the CRK gate, which consists of two QD-cavity systems (QD1 and QD2 gates) and an Rk (rotation k) operator with linearly optical devices, for the DQFT scheme. In order to demonstrate the CRk gate, let us suppose an input state of two photons A and B with the defined polarization, {|*R*〉, |*L*〉} ≡ {|0〉, |1〉}, as follows: ${|\phi \rangle }_{{\rm{in}}}={x}_{1}{|R\rangle }_{{\rm{A}}}^{1}{|R\rangle }_{{\rm{B}}}^{2}+{x}_{2}{|R\rangle }_{{\rm{A}}}^{1}{|L\rangle }_{{\rm{B}}}^{2}+{x}_{3}{|L\rangle }_{{\rm{A}}}^{1}{|R\rangle }_{{\rm{B}}}^{2}+{x}_{4}{|L\rangle }_{{\rm{A}}}^{1}{|L\rangle }_{{\rm{B}}}^{2}$. After a BS and an SF (path 2) on photon B, as described in Fig. 3, the transformed state, |*φ*〉_1_, is given by

9 $$\begin{array}{ccc}{|\phi \rangle }_{{\rm{in}}} & \mathop{\Rightarrow }\limits^{\mathrm{BS},\mathrm{SF}} & {|\phi \rangle }_{{\rm{1}}}=\frac{1}{\sqrt{2}}({x}_{1}{|R\rangle }_{{\rm{A}}}^{1}{|R\rangle }_{{\rm{B}}}^{1}+{x}_{2}{|R\rangle }_{{\rm{A}}}^{1}{|L\rangle }_{{\rm{B}}}^{1}+{x}_{3}{|L\rangle }_{{\rm{A}}}^{1}{|R\rangle }_{{\rm{B}}}^{1}+{x}_{4}{|L\rangle }_{{\rm{A}}}^{1}{|L\rangle }_{{\rm{B}}}^{1}+{x}_{1}{|R\rangle }_{{\rm{A}}}^{1}{|L\rangle }_{{\rm{B}}}^{2}+{x}_{2}{|R\rangle }_{{\rm{A}}}^{1}{|R\rangle }_{{\rm{B}}}^{2}+{x}_{3}{|L\rangle }_{{\rm{A}}}^{1}{|L\rangle }_{{\rm{B}}}^{2}+{x}_{4}{|L\rangle }_{{\rm{A}}}^{1}{|R\rangle }_{{\rm{B}}}^{2}).\end{array}$$

### [QD1 gate using reflection operator ${\hat{{\bf{R}}}}^{{\bf{1}}}$ (*ω* − *ω*_*c*_* = κ*/2)]

In QD1, the preparation of electron spin 1 state is in ${|{+}_{{\rm{e}}}\rangle }_{{\rm{1}}}$ where $|{\pm }_{{\rm{e}}}\rangle \equiv (|\uparrow \rangle \pm |\downarrow \rangle )/\sqrt{2}$. Then, the interaction, ${\hat{{\rm{R}}}}^{1}$, between QD1 and photons (A and B) is applied, and the procedure of switches (S1~S6) is performed in accordance with the time table shown in Fig. 3. After the state, |*φ*〉_1_, passes through SF on path 2 of photon B, the state is transformed into

10 $$\begin{array}{ccc}{|\phi \rangle }_{1}\mathop{\Rightarrow }\limits^{{\rm{Q}}{\rm{D}}1,\,{\rm{S}}{\rm{F}}}{|\phi \rangle }_{2} & = & \frac{1}{\sqrt{2}}(-{x}_{1}{|R\rangle }_{{\rm{B}}}^{1}{|R\rangle }_{{\rm{A}}}^{1}-{x}_{2}{|L\rangle }_{{\rm{B}}}^{2}{|R\rangle }_{{\rm{A}}}^{1}+{x}_{3}{|R\rangle }_{{\rm{B}}}^{2}{|L\rangle }_{{\rm{A}}}^{1}+{x}_{4}{|L\rangle }_{{\rm{B}}}^{1}{|L\rangle }_{{\rm{A}}}^{1})\otimes {|{-}_{{\rm{e}}}\rangle }_{1}\\  & - & \frac{i}{\sqrt{2}}({x}_{1}{|R\rangle }_{{\rm{B}}}^{2}{|R\rangle }_{{\rm{A}}}^{1}+{x}_{2}{|L\rangle }_{{\rm{B}}}^{1}{|R\rangle }_{{\rm{A}}}^{1}+{x}_{3}{|R\rangle }_{{\rm{B}}}^{1}{|L\rangle }_{{\rm{A}}}^{1}+{x}_{4}{|L\rangle }_{{\rm{B}}}^{2}{|L\rangle }_{{\rm{A}}}^{1})\otimes {|{+}_{{\rm{e}}}\rangle }_{1},\end{array}$$

where the reflection operator, ${\hat{{\rm{R}}}}^{1}$ (*ω* − *ω*_*c*_ = *κ*/2), in Eq. 8 applies to the above interaction of QD1 and photons. According to the measurement result regarding QD1, if the electron spin state is in ${|{-}_{{\rm{e}}}\rangle }_{{\rm{1}}}$, feed-forward (S_1_ and PF-R) is performed on photons A and B; otherwise, ${|{+}_{{\rm{e}}}\rangle }_{{\rm{1}}}$, it is not, as described in Fig. 3. Thus, the output state, |*φ*〉_O:1_, of QD1 gate is expressed as

11 $${|\phi \rangle }_{2}\mathop{\Rightarrow }\limits^{{\rm{f}}{\rm{e}}{\rm{e}}{\rm{d}}-{\rm{f}}{\rm{o}}{\rm{r}}{\rm{w}}{\rm{a}}{\rm{r}}{\rm{d}}\,{\rm{o}}{\rm{r}}\,{\rm{n}}{\rm{o}}{\rm{t}}}{|\phi \rangle }_{{\rm{O}}:1}={x}_{1}{|R\rangle }_{{\rm{B}}}^{2}{|R\rangle }_{{\rm{A}}}^{1}+{x}_{2}{|L\rangle }_{{\rm{B}}}^{1}{|R\rangle }_{{\rm{A}}}^{1}+{x}_{3}{|R\rangle }_{{\rm{B}}}^{1}{|L\rangle }_{{\rm{A}}}^{1}+{x}_{4}{|L\rangle }_{{\rm{B}}}^{2}{|L\rangle }_{{\rm{A}}}^{1}.$$

### [Rk operator and BS]

After the Rk operator on path 2 and BS is applied to photon B, as described in Fig. 3, the output state, |*φ*〉_O:1_, of QD1 gate is transformed into the state, |*φ*〉_3_, as follows:

12 $$\begin{array}{ccc}{|\phi \rangle }_{{\rm{O}}:1}\mathop{\Rightarrow }\limits^{{\rm{R}}{\rm{k}},\,{\rm{B}}{\rm{S}}}{|\phi \rangle }_{3} & = & \frac{1}{\sqrt{2}}({x}_{1}{|R\rangle }_{{\rm{B}}}^{1}{|R\rangle }_{{\rm{A}}}^{1}+{x}_{2}{|L\rangle }_{{\rm{B}}}^{1}{|R\rangle }_{{\rm{A}}}^{1}+{x}_{3}{|R\rangle }_{{\rm{B}}}^{1}{|L\rangle }_{{\rm{A}}}^{1}+{x}_{4}{e}^{2\pi i/{2}^{k}}{|L\rangle }_{{\rm{B}}}^{1}{|L\rangle }_{{\rm{A}}}^{1})\\  & + & \frac{1}{\sqrt{2}}(-{x}_{1}{|R\rangle }_{{\rm{B}}}^{2}{|R\rangle }_{{\rm{A}}}^{1}+{x}_{2}{|L\rangle }_{{\rm{B}}}^{2}{|R\rangle }_{{\rm{A}}}^{1}+{x}_{3}{|R\rangle }_{{\rm{B}}}^{2}{|L\rangle }_{{\rm{A}}}^{1}-{x}_{4}{e}^{2\pi i/{2}^{k}}{|L\rangle }_{{\rm{B}}}^{2}{|L\rangle }_{{\rm{A}}}^{1}).\end{array}$$

Subsequently, this state, |*φ*〉_3_, is injected into the QD2 gate in order to merge the split paths (1 and 2) of photon B.

### [QD2 gate using reflection operator ${\hat{{\bf{R}}}}^{{\bf{2}}}$ (*ω*−*ω*_*c*_* = *0)]

After the operation of the interaction, ${\hat{{\rm{R}}}}^{2}$, between QD2 and photon B with the preparation of electron spin 2 state as ${|{+}_{{\rm{e}}}\rangle }_{{\rm{2}}}$, the state, |*φ*〉_3_, is transformed into

13 $$\begin{array}{ccc}{|\phi \rangle }_{3}\mathop{\Rightarrow }\limits^{{\rm{Q}}{\rm{D}}2}{|\phi \rangle }_{4} & = & \frac{1}{\sqrt{2}}(-{x}_{1}{|R\rangle }_{{\rm{B}}}^{1}{|R\rangle }_{{\rm{A}}}^{1}+{x}_{2}{|L\rangle }_{{\rm{B}}}^{1}{|R\rangle }_{{\rm{A}}}^{1}-{x}_{3}{|R\rangle }_{{\rm{B}}}^{1}{|L\rangle }_{{\rm{A}}}^{1}+{x}_{4}{e}^{2\pi i/{2}^{k}}{|L\rangle }_{{\rm{B}}}^{1}{|L\rangle }_{{\rm{A}}}^{1})\otimes {|{-}_{{\rm{e}}}\rangle }_{2}\\  & + & \frac{1}{\sqrt{2}}(-{x}_{1}{|R\rangle }_{{\rm{B}}}^{2}{|R\rangle }_{{\rm{A}}}^{1}+{x}_{2}{|L\rangle }_{{\rm{B}}}^{2}{|R\rangle }_{{\rm{A}}}^{1}+{x}_{3}{|R\rangle }_{{\rm{B}}}^{2}{|L\rangle }_{{\rm{A}}}^{1}-{x}_{4}{e}^{2\pi i/{2}^{k}}{|L\rangle }_{{\rm{B}}}^{2}{|L\rangle }_{{\rm{A}}}^{1})\otimes {|{+}_{{\rm{e}}}\rangle }_{2},\end{array}$$

where the reflection operator, ${\hat{{\rm{R}}}}^{2}$ (*ω*−*ω*_*c*_ = 0), in Eq. 8 applies to the above interaction of QD2 and photon B. When measuring an electron spin 2 state in QD2, if the electron spin state is in ${|{-}_{{\rm{e}}}\rangle }_{{\rm{2}}}$, feed-forward (PF-R) is performed to photon A; otherwise, ${|{+}_{{\rm{e}}}\rangle }_{{\rm{1}}}$, feed-forward (S_2_, PF-R, and PF-L) is performed on photons A and B, as described in Fig. 3. Finally, after the input state, |*φ*〉_in_, passes through the CRk gate (QD1 and QD2 gates) in Fig. 3, the final state, |*φ*〉_fin_, can be given by

14 $${|\phi \rangle }_{{\rm{in}}}\mathop{\Rightarrow }\limits^{{\rm{CRk}}\,{\rm{gate}}}{|\phi \rangle }_{{\rm{fin}}}={x}_{1}{|R\rangle }_{{\rm{B}}}^{1}{|R\rangle }_{{\rm{A}}}^{1}+{x}_{2}{|L\rangle }_{{\rm{B}}}^{1}{|R\rangle }_{{\rm{A}}}^{1}+{x}_{3}{|R\rangle }_{{\rm{B}}}^{1}{|L\rangle }_{{\rm{A}}}^{1}+{x}_{4}{e}^{2\pi i/{2}^{k}}{|L\rangle }_{{\rm{B}}}^{1}{|L\rangle }_{{\rm{A}}}^{1}.$$

Consequently, the final state, |*φ*〉_fin_, which is applied to the CRk gate using two QD-cavity systems, is identical to the state, |*ϕ*〉_fin_, in Eq. 4, which is applied to the CRk operation (U_CRk_: swapping paths and operation of rotation k).

**Figure 4:**
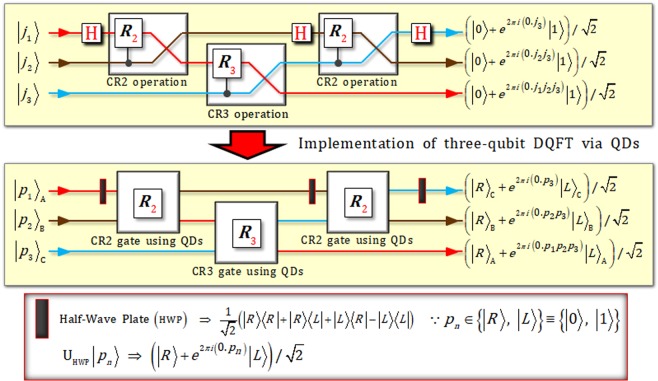
Three-qubit DQFT (the theoretical circuit of DQFT shown in Fig. 1), based on three CRk (two CR2 and a CR3) operations and three Hadamard operations, can be implemented in the three-photon DQFT scheme, which consists of three CPk (two CR2 and a CR3) gates using the QD-cavity systems, and three HWPs. The HWP, which can rotate the polarization of a photon, performs the Hadamard operation.

Now, using a simple example in Fig. 4, we show the implementation of the three-qubit DQFT scheme by directly utilizing the proposed CRk gates via the QD-cavity systems. As shown in Fig. 4, we can realize the theoretical circuit of the three-qubit DQFT to three-photon DQFT scheme employing CRk gates (QD-cavity systems) and HWPs by arranging our CRk gates in the manner shown in Fig. 3. Thus, for the scalability of the multi-photon DQFT scheme (for the implementation of multi-qubit DQFT), the structure of the three-photon DQFT scheme can be generalized (or expanded) to realize the multi-photon DQFT scheme by the composition of the CRk (*k* = 2, …, *t*) gates and HWPs.

The structure of our DQFT scheme, which consists of CRk gates using QDs, is based on the theoretical circuit of DQFT in Fig. 1. When our scheme is expended to multi-photon DQFT scheme, the method of expansion is identical with the procedure of the theoretical circuit of multi-qubit DQFT algorithm, as described in Fig. 4. Thus, we can calculate the complexity^2^ about how many gates our DQFT scheme is utilizing on *t* photons, as follows: The total of *t* gates (a HWP and *t* − 1 CRk gates) is used on the first photon. And the total of *t* − 1 gates (a HWP and *t* − 2 CRk gates) is used on the second photon. Finally, the *t*th photon only utilizes one gate (a HWP). In our DQFT scheme, we can confirm that the number of gates for DQFT is as *t*(*t* + 1)/2 = *t* + (*t* − 1) + ⋅⋅⋅ + 2 + 1. Consequently, our DQFT scheme provides a Θ(*t*^2^) algorithm for the operation of DQFT on *t* photons. Furthermore, compared with the classical best algorithm as fast Fourier transform (FFT)^2^, which provides a Θ(*t*2^*t*^) algorithm for the discrete Fourier transform, our DQFT scheme on quantum computer is more efficient than FFT, which requires exponentially more operations, on classical computer.

So far, we have proposed the use of the CRk gate, using the QD-cavity systems, to feasibly implement the CRk (controlled-rotation k) operation for the optical DQFT scheme with high efficiency and reliable performance. In the optical DQFT, our CRk gate with two QD-cavity systems (QD1 and QD2 gates) is the essential element with the stable storage of quantum information and the actual interaction between the photon and QD. Thus, for the experimental realization of our CRk gate, we should analyze the interaction of the QD-cavity system (QD within a single-sided cavity) under vacuum noise, sideband leakage, and absorption, in practice.

## Analysis of QD-Cavity System under Vacuum Noise and Sideband Leakage

In our CRk gate, in order to implement a controlled-rotation k (CRk) operation, the critical component is the interaction of the QD-cavity system inducing a difference in reflectance |*r*_h_| [|*r*_0_|] with phase shift *ϕ*_rh_ [*ϕ*_r0_] from the reflection coefficient *R*_h_ [*R*_0_] shown in Eq. 6 with respect to the hot [cold] cavity. Thus, the interaction between a photon and an electron spin state in QD should be analyzed to quantify the efficiency and reliable performance of the QD-cavity system under vacuum noise, *N*(*ω*), for operation of the QD-dipole and leaky modes, *S*(*ω*) (sideband leakage and absorption)^40,42,53–56^. For this analysis, we introduce the Jaynes-Cummings Hamiltonian (*H*_JC_) in the rotating frame at the input field (${\hat{{b}}}_{{\rm{in}}}$) frequency.

15 $${H}_{{\rm{JC}}}=\hslash ({\omega }_{c}-\omega ){\hat{{a}}}^{+}\hat{{a}}+\hslash ({\omega }_{{{\rm{X}}}^{-}}-\omega ){\hat{\sigma }}_{+}{\hat{\sigma }}_{-}+i\hslash g({\hat{\sigma }}_{+}\hat{{a}}-{\hat{{a}}}^{+}{\hat{\sigma }}_{-})+\hslash \int d\omega ^{\prime} \omega ^{\prime} {\hat{{b}}}^{+}\hat{{b}}+\frac{\hslash }{\sqrt{2\pi }}\int d\omega ^{\prime} \sqrt{\kappa }(\hat{{a}}{\hat{{b}}}^{+}+{\hat{{a}}}^{+}\hat{{b}}).$$

Using the solutions of the Heisenberg equations of motion (Bloch equation with damping, *γ*), we can calculate the quantum Langevin equations of a cavity field operator, $\hat{{a}}$, a dipole operator, ${\hat{\sigma }}_{-}$, of X^−^, and the input-output relations with vacuum noise, *N*(*ω*), and leaky modes, *S*(*ω*), as follows:

16 $$\begin{array}{ccc}\frac{d\hat{a}}{dt} & = & -\frac{i}{\hslash }[\hat{a},\,{H}_{{\rm{J}}{\rm{C}}}]=-[i({\omega }_{c}-\omega )+\frac{\kappa }{2}+\frac{{\kappa }_{s}}{2}]\hat{a}-g{\hat{\sigma }}_{-}-\sqrt{\kappa }{\hat{b}}_{{\rm{i}}{\rm{n}}}-\sqrt{{\kappa }_{s}}{\hat{S}}_{{\rm{i}}{\rm{n}}},\\ \frac{d{\hat{\sigma }}_{-}}{dt} & = & -\frac{i}{\hslash }[{\hat{\sigma }}_{-},\,{H}_{{\rm{J}}{\rm{C}}}]=-[i({\omega }_{{{\rm{X}}}^{-}}-\omega )+\frac{\gamma }{2}]{\hat{\sigma }}_{-}-g{\hat{\sigma }}_{Z}\hat{a}+\sqrt{\gamma }{\hat{\sigma }}_{Z}\hat{N},\\ \frac{d{\hat{\sigma }}_{Z}}{dt} & = & \frac{d({\rho }_{22}-{\rho }_{11})}{dt}=0,\\ {\hat{b}}_{{\rm{o}}{\rm{u}}{\rm{t}}} & = & {\hat{b}}_{{\rm{i}}{\rm{n}}}+\sqrt{\kappa }\hat{a},\,{\hat{S}}_{{\rm{o}}{\rm{u}}{\rm{t}}}={\hat{S}}_{{\rm{i}}{\rm{n}}}+\sqrt{\kappa }\hat{a},\end{array}$$

where ${\hat{S}}_{{\rm{in}}}({\hat{S}}_{{\rm{out}}})$ is the input (output) field operator from leaky modes due to sideband leakage and absorption in the cavity mode, and $\hat{N}$ is the vacuum noise operator for ${\hat{\sigma }}_{-}$. In Sec. 3.1, we assumed the approximation of weak excitation with the ground state, $\langle {\hat{\sigma }}_{Z}\rangle =-1$ (no saturation), in the QD for the steady state^42,45,46,50–52^. Based on this condition and Eq. 16, the output field operator, ${\hat{{b}}}_{{\rm{out}}}$, can be given by

17 $${\hat{{b}}}_{{\rm{out}}}=R(\omega ){\hat{{b}}}_{{\rm{in}}}+N(\omega )\hat{N}+S(\omega ){\hat{S}}_{{\rm{in}}}.$$

Here, we consider the vacuum noise, *N*(*ω*), and leaky mode, *S*(*ω*) (sideband leakage and absorption) in the interaction of the QD-cavity, and contrast it with the case presented in Eq. 5 (the ideal case: ${\hat{{b}}}_{{\rm{out}}}=R(\omega ){\hat{{b}}}_{{\rm{in}}}$). Therefore, the coefficients of noise, *N*(*ω*), and leakage, *S*(*ω*), can be calculated with ${\omega }_{{{\rm{X}}}^{-}}={\omega }_{c}$, as follows:

18 $$\begin{array}{rcl}N(\omega ) & = & \frac{\sqrt{\gamma \kappa }g}{[i({\omega }_{c}-\omega )+\gamma /2][i({\omega }_{c}-\omega )+\kappa /2+{\kappa }_{s}/2]+{g}^{2}},\\ S(\omega ) & = & \frac{-\sqrt{{\kappa }_{s}\kappa }[i({\omega }_{c}-\omega )+\gamma /2]}{[i({\omega }_{c}-\omega )+\gamma /2][i({\omega }_{c}-\omega )+\kappa /2+{\kappa }_{s}/2]+{g}^{2}},\end{array}$$

where the reflection coefficient, *R*(*ω*), is obtained from Eq. 5. Then, we can acquire the noise rate-phase shift (|*n*_h_| - *ϕ*_nh_) [|*n*_0_| - *ϕ*_n0_], as well as the leakage rate-phase shift (|*s*_h_| - *ϕ*_sh_) [|*s*_0_| - *ϕ*_s0_] from the coefficients of noise, ${N}_{{\rm{h}}}\equiv |{n}_{{\rm{h}}}|{e}^{i{\phi }_{{\rm{nh}}}}$ $[{N}_{{\rm{0}}}\equiv |{n}_{{\rm{0}}}|{e}^{i{\phi }_{{\rm{n0}}}}]$, and leakage, ${S}_{{\rm{h}}}\equiv |{s}_{{\rm{h}}}|{e}^{i{\phi }_{{\rm{sh}}}}$ $[{S}_{{\rm{0}}}\equiv |{s}_{{\rm{0}}}|{e}^{i{\phi }_{{\rm{s0}}}}]$, corresponding to the (hot: *g* ≠ 0) [cold: *g* = 0] cavity.

**Figure 5:**
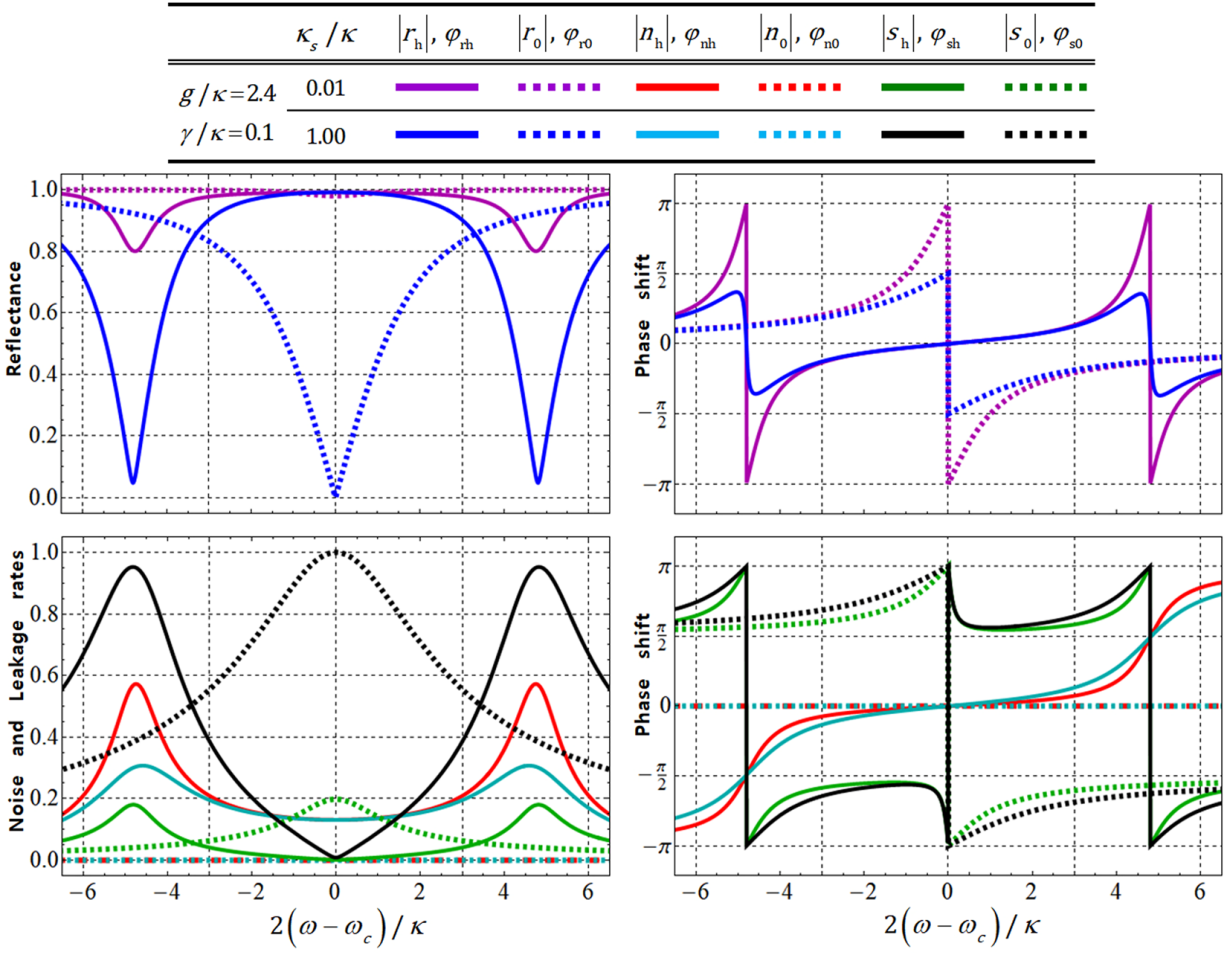
Reflectances (|*r*_h_|, |*r*_0_|), noise rates (|*n*_h_|, |*n*_0_|), leakage rates (|*s*_h_|, |*s*_0_|), and phase shifts (*ϕ*_rh_, *ϕ*_r0_, *ϕ*_nh_, *ϕ*_n0_, *ϕ*_sh_, *ϕ*_s0_) for the detuning frequency, 2(*ω* − *ω*_*c*_)/*κ* in terms of the differences in the side-leakage rates (*κ*_*s*_/*κ* = 0.01 and *κ*_*s*_/*κ* = 1.00). In these plots, the experimental parameters (the coupling strength and decay rate of X^−^) are fixed at *g*/*κ* = 2.4 and *γ*/*κ* = 0.1 [*g* ≫ (*γ*, *κ*)] with ${\omega }_{{{\rm{X}}}^{-}}={\omega }_{c}$.

The graphs shown in Fig. 5 represent the values of the reflectances (|*r*_h_|, |*r*_0_|), noise rates (|*n*_h_|, |*n*_0_|), leakage rates (|*s*_h_|, |*s*_0_|), and phase shifts (*ϕ*_rh_, *ϕ*_r0_, *ϕ*_nh_, *ϕ*_n0_, *ϕ*_sh_, *ϕ*_s0_) for the detuning frequency as 2(*ω* − *ω*_*c*_)/*κ* in terms of the differences in the side-leakage rates (*κ*_*s*_/*κ* = 0.01 and 1.00) with *g*/*κ* = 2.4, *γ*/*κ* = 0.1, and ${\omega }_{{{\rm{X}}}^{-}}={\omega }_{c}$. As illustrated in Fig. 5, if we take the small side-leakage, *κ*_*s*_/*κ* = 0.01, with *g*/*κ* = 2.4 and *γ*/*κ* = 0.1, the values of the reflectances, noise rates, leakage rates, and phase shifts can be respectively acquired as |*r*_h_| ≈ 1 (≈1), |*r*_0_| ≈ 1 (≈1), |*n*_h_| ≈ 0.1 (≈0.1), |*n*_0_| = 0 (=0), |*s*_h_| ≈ 0 (≈0), |*s*_0_| ≈ 0.1 (≈0.2), and *ϕ*_rh_ ≈ 0 (≈0), *ϕ*_r0_ ≈ −*π*/2 (=*π*), *ϕ*_nh_ ≈ 0 (≈0), *ϕ*_n0_ = 0 ( = 0), *ϕ*_sh_ ≈ 11*π*/20 (=*π*), *ϕ*_s0_ ≈ −3*π*/4 (=*π*) in the adjusted frequencies as *ω* − *ω*_*c*_ = *κ*/2 (*ω* − *ω*_*c*_ = 0). Based on these values, we can confirm that the affections of the vacuum noise, *N*(*ω*), and leaky modes, *S*(*ω*) (sideband leakage and absorption) will be small (ignored), due to the extremely small values of noise rates (|*n*_h_|, |*n*_0_|) and leakage rates (|*s*_h_|, |*s*_0_|) for *g*/*κ* = 2.4 and *γ*/*κ* = 0.1, respectively, with *κ*_*s*_/*κ* = 0.01 (small side-leakage rate)^40,42,53–56^.

In addition, for the quantifying efficiency and performance of the QD-cavity system, we should modify the reflection operator, $\hat{{\rm{R}}}(\omega )$ in Eq. 7, into the practical reflection operator, ${\hat{{\rm{R}}}}_{{\rm{p}}}(\omega )$, including to the affections of the vacuum noise, *N*(*ω*), and leaky modes, *S*(*ω*) (sideband leakage and absorption), as follows:

19 $$\begin{array}{rcl}{\hat{{\rm{R}}}}_{{\rm{p}}}(\omega ) & = & [|{r}_{{\rm{h}}}(\omega )|{e}^{i{\phi }_{{\rm{rh}}}(\omega )}+|{n}_{{\rm{h}}}(\omega )|{e}^{i{\phi }_{{\rm{nh}}}(\omega )}+|{s}_{{\rm{h}}}(\omega )|{e}^{i{\phi }_{{\rm{sh}}}(\omega )}]\\  &  & \times \,(|R\rangle \langle R|\otimes |\downarrow \rangle \langle \downarrow |+|L\rangle \langle L|\otimes |\uparrow \rangle \langle \uparrow |)\\  &  & +\,[|{r}_{0}(\omega )|{e}^{i{\phi }_{{\rm{r0}}}(\omega )}+|{s}_{{\rm{0}}}(\omega )|{e}^{i{\phi }_{{\rm{s0}}}(\omega )}](|R\rangle \langle R|\otimes |\uparrow \rangle \langle \uparrow |+|L\rangle \langle L|\otimes |\downarrow \rangle \langle \downarrow |),\end{array}$$

where ${N}_{{\rm{0}}}(\omega )\equiv |{n}_{{\rm{0}}}(\omega )|\exp [i{\phi }_{{\rm{n0}}}(\omega )]=0$ (cold cavity: *g* = 0), as shown in Eq. 18. Therefore, we can analyze the efficiencies and performances of the QD-cavity systems (QD1 and QD2 gates) to quantify the fidelity values of the output states from the reflection operators, ${\hat{{\rm{R}}}}^{1}$ (*ω* − *ω*_*c*_ = *κ*/2) and ${\hat{{\rm{R}}}}^{2}$ (*ω* − *ω*_*c*_ = 0) in Eq. 8 (ideal case), and the reflection operator, ${\hat{{\rm{R}}}}_{{\rm{p}}}$ in Eq. 19 (practical case). When the input state is assumed to be $(|R\rangle +|L\rangle )/\sqrt{2}\otimes (|\uparrow \rangle +|\downarrow \rangle )/\sqrt{2}$, the ideal output states, $|{\psi }_{{\rm{ID}}}^{1}\rangle (\mathrm{QD1}:\,\omega -{\omega }_{c}=\kappa /2)$ and $|{\psi }_{{\rm{ID}}}^{2}\rangle (\mathrm{QD2}:\,\omega -{\omega }_{c}=0)$, from Eq. 8, and a practical output state, |*ψ*_PR_〉, from Eq. 19, can be given by

20 $$\begin{array}{rcl}|{\psi }_{{\rm{ID}}}^{1}\rangle  & = & \frac{1}{\sqrt{2}}[\frac{1}{\sqrt{2}}(|R\rangle |\downarrow \rangle +|L\rangle |\uparrow \rangle )-\frac{i}{\sqrt{2}}(|R\rangle |\uparrow \rangle +|L\rangle |\downarrow \rangle )],\\ |{\psi }_{{\rm{ID}}}^{2}\rangle  & = & \frac{1}{\sqrt{2}}[\frac{1}{\sqrt{2}}(|R\rangle |\downarrow \rangle +|L\rangle |\uparrow \rangle )-\frac{1}{\sqrt{2}}(|R\rangle |\uparrow \rangle +|L\rangle |\downarrow \rangle )],\\ |{\psi }_{{\rm{PR}}}\rangle  & = & \frac{1}{\sqrt{{\rm{N}}}}[\frac{({R}_{{\rm{h}}}+{N}_{{\rm{h}}}+{S}_{{\rm{h}}})}{\sqrt{2}}(|R\rangle |\downarrow \rangle +|L\rangle |\uparrow \rangle )+\frac{({R}_{{\rm{0}}}+{S}_{{\rm{0}}})}{\sqrt{2}}(|R\rangle |\uparrow \rangle +|L\rangle |\downarrow \rangle )],\end{array}$$

where N = |*R*_h_ + *N*_h_ + *S*_h_|^2^ + |*R*_0_ + *S*_0_|^2^. Then, we can calculate the fidelities F_1_ (QD1 gate) between $|{\psi }_{{\rm{ID}}}^{1}\rangle$ and |*ψ*_PR_〉, and F_2_ (QD2 gate) between $|{\psi }_{{\rm{ID}}}^{2}\rangle$ and |*ψ*_PR_〉 as follows:

21 $$\begin{array}{c}{{\rm{F}}}_{{\rm{1}}}\equiv |\sqrt{\langle {\psi }_{{\rm{PR}}}|{\psi }_{{\rm{ID}}}^{1}\rangle \langle {\psi }_{{\rm{ID}}}^{1}|{\psi }_{{\rm{PR}}}\rangle }|=\frac{1}{\sqrt{2{\rm{N}}}}|\sqrt{{|({R}_{{\rm{h}}}+{N}_{{\rm{h}}}+{S}_{{\rm{h}}})-i({R}_{{\rm{0}}}+{S}_{{\rm{0}}})|}^{2}}|,\\ {{\rm{F}}}_{{\rm{2}}}\equiv |\sqrt{\langle {\psi }_{{\rm{PR}}}|{\psi }_{{\rm{ID}}}^{2}\rangle \langle {\psi }_{{\rm{ID}}}^{2}|{\psi }_{{\rm{PR}}}\rangle }|=\frac{1}{\sqrt{2{\rm{N}}}}|\sqrt{{|({R}_{{\rm{h}}}+{N}_{{\rm{h}}}+{S}_{{\rm{h}}})-({R}_{{\rm{0}}}+{S}_{{\rm{0}}})|}^{2}}|.\end{array}$$

**Figure 6:**
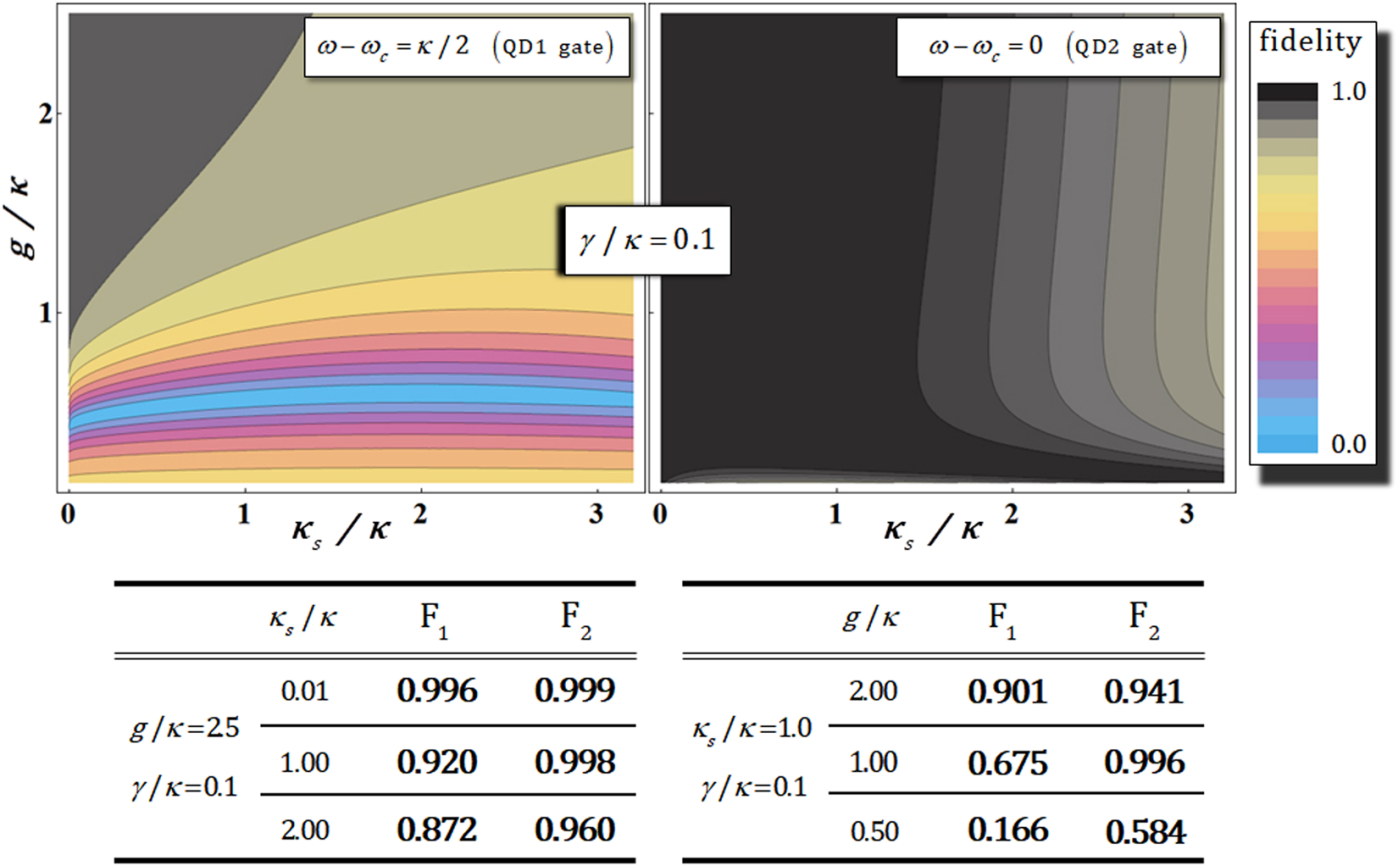
Fidelities, F_1_ (QD1 gate: *ω* − *ω*_*c*_ = *κ*/2) and F_2_ (QD2 gate: *ω* − *ω*_*c*_ = 0) of the output states for the side-leakage rate *κ*_*s*_/*κ* and coupling strength *g*/*κ* with *γ*/*κ* = 0.1 and ${\omega }_{{{\rm{X}}}^{-}}={\omega }_{c}$ under vacuum noise, *N*(*ω*), for the operation of the QD-dipole and leaky modes, *S*(*ω*) (sideband leakage and absorption). In the tables, the values of fidelities F_1_ and F_2_ are listed for the differences in *κ*_*s*_/*κ* with *g*/*κ* = 2.5, and also the differences in *g*/*κ* with *κ*_*s*_/*κ* = 1.0.

The graphs and tables in Fig. 6 show the distributions and values of fidelities, F_1_ (QD1 gate: $|{\psi }_{{\rm{ID}}}^{1}\rangle \leftrightarrow |{\psi }_{{\rm{PR}}}\rangle$) and F_2_ (QD2 gate: $|{\psi }_{{\rm{ID}}}^{{\rm{2}}}\rangle \leftrightarrow |{\psi }_{{\rm{PR}}}\rangle$), according to the differences in the side-leakage rates, *κ*_*s*_/*κ*, as well as the coupling strength, *g*/*κ*, with *γ*/*κ* = 0.1 under vacuum noise, *N*(*ω*), and sideband leakage and absorption, *S*(*ω*). As described in Fig. 6, when we take the experimental parameters as the strong coupling strength, *g* ≫ (*κ*, *γ*), and the small side-leakage rate, *κ*_*s*_ ≪ *κ*, with fixed *γ*/*κ* = 0.1, two fidelities (F_1_ and F_2_) are approaching 1 despite the affection of vacuum noise, *N*(*ω*), and sideband leakage, *S*(*ω*). Thus, we can conclude that the influences of the noise rate, leakage rate, and phase shifts in Eq. 18 will be ignored if the magnitude of coupling strength (*g*/*κ*) increases, and if the side-leakage rate (*κ*_*s*_/*κ*), decreases in the QD-cavity system.

Consequently, by analysis of the efficiency and performance of the QD-cavity system in terms of the fidelities, F_1_ and F_2_, from the reflection operator, ${\hat{{\rm{R}}}}_{{\rm{p}}}(\omega )$, in Eq. 19, we demonstrate that the experimental implementation of our CRk gate using QD1 and QD2 gates is feasible when there is a strong coupling strength, *g* ≫ (*κ*, *γ*), and the small side-leakage rate, *κ*_*s*_ ≪ *κ*.

## Conclusions

Thus far, we have designed an optical CRk gate which can realize the controlled-rotation k (CRk) operation between two photons, using QD confined in a single-sided optical cavity. The theoretical circuit of DQFT can be directly implemented using the proposed CRk gates. In addition, we can achieve scalability in the multi-photon DQFT scheme to simply arrange the CRk (*k* = 2, ‥, *t*) gates and HWPs (i.e. three-photon DQFT scheme in Sec. 3.2) for applicable multi-qubit DQFT. Thus, in order to successfully design a practical CRk gate in the structure of DQFT, we also analyzed the efficiency and performance of the interaction in the QD-cavity system to quantify the fidelities of the QD1 and QD2 gates (components of a CRk gate), and also demonstrated a method, using strong coupling strength and small side-leakage rate as *g* ≫ (*κ*, *γ*) and *κ*_*s*_ ≪ *κ*, to improve the robustness against the affection of the vacuum noise, *N*(*ω*), for the operation of the QD-dipole and leaky modes, *S*(*ω*) (sideband leakage and absorption)^40,42,53–56^, as described in Sec. 4.

In the previous works of QFT (including DQFT) schemes, the various physical resources have been employed for the operation of QFT. Though the QFT schemes^21,22^, which are implemented using the linearly optical devices, has the advantage to realize the operation of QFT with the experimental simplicity, the performances (accuracies) of QFT schemes are probabilistic (as less than 35%)^21,22^. Also, the affection of the inaccuracy influences the entire performance of quantum algorithms because QFT or DQFT is a main subroutine of quantum algorithms. Therefore, many researchers have proposed the QFT schemes via the nonlinearly optical devices, such as cross-Kerr nonlinearities (XKNLs)^17,25^, and optical cavities^15,27,31–33^. However, the experimental realization of strong XKNL is still a big challenge^75^. And the decoherence effect, which can occurs the evolution of the mixed state after homodyne measurement, is also unavoidable when a coherent state is transmitted through a fiber in practice^17,25,36,69^. From this point of view, well-isolated qubits might not necessarily suffer from the same decoherence effects in Kerr medium. The optical cavity system, which is one of the candidates for the QFT schemes, has recently attracted extensive attention. Weinstein *et al*.^17^ and Scully *et al*.^31^ have designed to implement the operations of QFT using the Ion trap and cavity QED. But the drawback of these schemes is to require a large number of qubits for the operation of QFT. And the QFT schemes^15,32,33^ based on the distant atoms trapped in separate cavities could be restrictively utilized for the one-way quantum computing, due to the trapped atoms used as qubits.

Compared with the previous schemes^15,17,21,22,25,27,31–33^, our DQFT scheme consists of the QD-cavity systems (nonlinearly optical devices) to implement the CRk operations as the basic modules for the deterministic performance and simple expansion into multi-qubit DQFT. Moreover, in our scheme, the flying photons play the roles of qubits transferring quantum information, and QDs are the ancillary systems to efficiently perform the CRk operations for the applications of various quantum computations and algorithms.

Also, In view of the experimental realization, for the high fidelity (condition of *g* ≫ (*κ*, *γ*) and *κ*_*s*_ ≪ *κ*) of the interaction between a photon and the QD-cavity system, researchers have proposed a variety of experimental techniques, including the following: Rosenblum *et al*.^68^ obtained the coupling strength as *g*/(*κ* + *κ*_*s*_) ≈ 0.5 in a micropillar cavity (d = 1.5 μm) with a quality factor Q = 8800, and for Q = 40000, the coupling strength could be increased to *g*/(*κ* + *κ*_*s*_) ≈ 2.4^74^. Furthermore, Arnold *et al*.^76^ enhanced the quality factor, Q = 215000 (*κ* ≈ 6.2 μeV), in order to acquire a small side-leakage rate. Reitzensteina *et al*.^77^ demonstrated two methods (the etching process and improving the sample growth) to reduce the side-leakage rate, *κ*_*s*_/*κ*, in an In_0.6_Ga_0.4_As optical cavity having *g*/(*κ* + *κ*_*s*_) ≈ 2.4 and Q = 40000. Thus, the QD-cavity systems in our CRk gate can be reliably operated with high fidelity, according to the results of our analysis presented in Sec. 4. Moreover, in order to ensure the reliable interaction between a flying photon and stationary qubit (electron spin in QD), the initialization (electron spin-superposition state) and manipulation of the spin state can be prepared by optical pumping or optical cooling^78^, and can be acquired using pulsed magnetic resonance techniques, nanosecond microwave pulses, or picosecond/femtosecond optical pulses^79–82^.

Therefore, we obtain the interaction of the CRk gate, using the QD-cavity system, with high fidelity for the DQFT scheme. Consequently, according to our analysis results, in practice (under vacuum noise and sideband leakage), we can obtain high efficiency and reliable performance of the interaction between a photon and QD within a single-sided optical cavity (CRk gate). Our results also indicate that our DQFT scheme using CRk gates can be experimentally feasible, as well as scalable for multi-qubit DQFT.

## References

[1] Kitaev, A. Quantum measurements and the Abelian stabilizer problem. arxiv quant-ph/9511026 (1995).

[2] Nielsen, M. A. & Chuang, I. L. *Quantum Computation and Quantum Information*. (Cambridge University Press, 2000).

[3] Danilin S (2018). Quantum-enhanced magnetometry by phase estimation algorithms with a single artificial atom. npj Quantum. Inf..

[4] Shor, P. W. Algorithms for quantum computation: Discrete logarithms and factoring. In Proceedings, 35th Annual Symposium on Foundations of Computer Science. 124 (1994).

[5] Vandersypen LMK (2000). Experimental Realization of an Order-Finding Algorithm with an NMR Quantum Computer. Phys. Rev. Lett..

[6] Lanyon BP (2007). Experimental Demonstration of a Compiled Version of Shor’s Algorithm with Quantum Entanglement. Phys. Rev. Lett..

[7] Peng WC (2019). Factoring larger integers with fewer qubits via quantum annealing with optimized parameters. Sci. China-Phys. Mech. Astron..

[8] Mosca M, Zalka C (2004). Exact quantum Fourier transforms and discrete logarithm algorithms. Int. J. Quantum Inf..

[9] Song, S. Y. Quantum Computing for Discrete Logarithms. *Quantum Computational Number Theory*. (Springer, Cham, 121, 2015).

[10] Michele, M. & Ekert, A. The hidden subgroup problem and eigenvalue estimation on a quantum computer. *Quantum Computing and Quantum Communications*. (Springer, Berlin, Heidelberg, 174, 1999).

[11] Jozsa R (2001). Quantum factoring, discrete logarithms, and the hidden subgroup problem. Computing in science & engineering.

[12] Gonçalves DN, Fernandes TD, Cosme CMM (2017). An efficient quantum algorithm for the hidden subgroup problem over some non-abelian groups. TEMA.

[13] Chiaverini J (2005). Implementation of the Semiclassical Quantum Fourier Transform in a Scalable System. Science.

[14] Wang HF, Shao XQ, Zhao YF, Zhang S, Yeon KH (2010). Protocol and quantum circuit for implementing the N-bit discrete quantum Fourier transform in cavity QED. J. Phys. B.

[15] Wang HF, Zhu AD, Zhang S, Yeon KH (2011). Simple implementation of discrete quantum Fourier transform via cavity quantum electrodynamics. New J. phys..

[16] Obada ASF, Hessian HA, Mohamed ABA, Homid AH (2013). Implementing discrete quantum Fourier transform via superconducting qubits coupled to a superconducting cavity. J. Opt. Soc. Am. B.

[17] Heo J, Kang MS, Hong CH, Yang H, Choi SG (2016). Discrete quantum Fourier transform using weak cross-Kerr nonlinearity and displacement operator and photon-number-resolving measurement under the decoherence effect. Quantum Inf. Process..

[18] Zhou SS, Loke T, Izaac JA, Wang JB (2017). Quantum Fourier transform in computational basis. Quantum Inf. Process..

[19] Li HS, Fan P, Xia HY, Song S, He X (2018). The quantum Fourier transform based on quantum vision representation. Quantum Inf. Process..

[20] Bhattacharya N, van den Heuvell HBL, Spreeuw R (2002). Implementation of Quantum Search Algorithm using Classical Fourier Optics. Phys. Rev. Lett..

[21] Mohseni M, Lundeen J, Resch K, Steinberg A (2003). Experimental Application of Decoherence-Free Subspaces in an Optical Quantum-Computing Algorithm. Phys. Rev. Lett..

[22] Barak R, Ben-aryeh Y (2007). Quantum fast Fourier transform and quantum computation by linear optics. J. Opt. Soc. Am. B.

[23] Loock PV (2008). Hybrid quantum computation in quantum optics. Phys. Rev. A.

[24] Lin Q, He B (2010). Addendum to “Single-photon logic gates using minimum resources”. Phys. Rev. A.

[25] Dong L, Xiu XM, Shen HZ, Gao YJ, Yi XX (2013). Quantum Fourier transform of polarization photons mediated by weak cross-Kerr nonlinearity. J. Opt. Soc. Am. B.

[26] Cirac J, Zoller P (1995). Quantum Computations with Cold Trapped Ions. Phys. Rev. Lett..

[27] Weinstein Y, Pravia M, Fortunato E (2001). Implementation of the Quantum Fourier Transform. Phys. Rev. Lett..

[28] Gulde S (2003). Implementation of the Deutsch–Jozsa algorithm on an ion-trap quantum computer. Nature.

[29] Fujiwara S, Hasegawa S (2005). General method for realizing the conditional phase-shift gate and a simulation of Grover’s algorithm in an ion-trap system. Phys. Rev. A.

[30] Niskanen A, Vartiainen J, Salomaa M (2003). Optimal Multiqubit Operations for Josephson Charge Qubits. Phys. Rev. Lett..

[31] Scully M, Zubairy M (2002). Cavity QED implementation of the discrete quantum Fourier transform. Phys. Rev. A.

[32] Wang HF, Zhang S, Yeon KH (2008). Implementing Quantum Discrete Fourier Transform by Using Cavity Quantum Electrodynamics: J. Korean Phys. Soc..

[33] Wang HF, Zhang S, Zhu AD, Yeon KH (2012). Fast and effective implementation of discrete quantum Fourier transform via virtual-photon-induced process in separate cavities. J. Opt. Soc. Am. B.

[34] Nemoto K, Munro WJ (2004). Nearly Deterministic Linear Optical Controlled-NOT Gate. Phys. Rev. Lett..

[35] Lin Q, Li J (2009). Quantum control gates with weak cross-Kerr nonlinearity. Phys. Rev. A.

[36] Heo J, Hong CH, Yang HJ, Hong JP, Choi SG (2017). Analysis of optical parity gates of generating Bell state for quantum information and secure quantum communication via weak cross-Kerr nonlinearity under decoherence effect. Quantum Inf. Process..

[37] Xiu XM (2018). Constructing the nearly deterministic Toffoli polarization gate with the spatial degree of freedom based on weak cross-Kerr nonlinearities. Opt. Commun..

[38] Chow JM (2012). Universal Quantum Gate Set Approaching Fault-Tolerant Thresholds with Superconducting Qubits. Phys. Rev. Lett..

[39] Kim H, Bose R, Shen TC, Solomon GS, Waks E (2013). A quantum logic gate between a solid-state quantum bit and a photon. Nat. Photonics.

[40] Heo J (2017). Implementation of controlled quantum teleportation with an arbitrator for secure quantum channels via quantum dots inside optical cavities. Sci. Rep..

[41] Song GZ, Yang GJ, Zhang M (2017). Compact quantum gates for hybrid photon–atom systems assisted by Faraday rotation. Quantum. Inf. Process..

[42] Hong CH, Heo J, Kang MS, Jang J, Yang HJ (2018). Optical scheme for generating hyperentanglement having photonic qubit and time-bin via quantum dot and cross-Kerr nonlinearity. Sci. Rep..

[43] Kang MS, Heo J, Choi SG, Sung M, Han SW (2019). Implementation of SWAP test for two unknown states in photons via cross-Kerr nonlinearities under decoherence effect. Sci. Rep..

[44] Imamoglu A (1999). Quantum Information Processing Using Quantum Dot Spins and Cavity QED. Phys. Rev. Lett..

[45] Hu CY, Young A, O’Brien JL, Munro WJ, Rarity JG (2008). Giant optical Faraday rotation induced by a single-electron spin in a quantum dot: applications to entangling remote spins via a single photon. Phys. Rev. B.

[46] Hu CY, Munro WJ, O’Brien JL, Rarity JG (2009). Proposed entanglement beam splitter using a quantum-dot spin in a double-sided optical microcavity. Phys. Rev. B.

[47] Gao WB (2013). Quantum teleportation from a propagating photon to a solid-state spin qubit. Nat. Commun..

[48] Luo MX, Wang X (2014). Parallel photonic quantum computation assisted by quantum dots in one-side optical microcavities. Sci. Rep..

[49] Kuhlmann AV (2015). Transform-limited single photons from a single quantum dot. Nat. Commun..

[50] Heo J, Kang MS, Hong CH, Choi SG, Hong JP (2017). Constructions of secure entanglement channels assisted by quantum dots inside single-sided optical cavities. Opt. Commun..

[51] Hu CY (2017). Photonic transistor and router using a single quantum-dot confined spin in a single-sided optical microcavity. Sci. Rep..

[52] Heo J, Kang MS, Hong CH, Choi SG, Hong JP (2017). Scheme for secure swapping two unknown states of a photonic qubit and an electron-spin qubit using simultaneous quantum transmission and teleportation via quantum dots inside single-sided optical cavities. Phys. Lett. A.

[53] Waks E, Vuckovic J (2006). Dipole Induced Transparency in Drop-Filter Cavity-Waveguide Systems. Phys. Rev. Lett..

[54] Wang B, Duan LM (2007). Implementation scheme of controlled SWAP gates for quantum fingerprinting and photonic quantum computation. Phys. Rev. A.

[55] Li T, Yang GJ, Deng FG (2016). Heralded quantum repeater for a quantum communication network based on quantum dots embedded in optical microcavities. Phys. Rev. A.

[56] Hong C, Heo J, Kang MS, Jang J, Yang HJ (2019). Scheme for encoding single logical qubit information into three-photon decoherence-free states assisted by quantum dots. Quantum Inf. Process..

[57] Petta JR (2005). Coherent Manipulation of Coupled Electron Spins in Semiconductor Quantum Dots. Science.

[58] Greilich A (2006). Mode locking of electron spin coherences in singly charged quantum dots. Science.

[59] Xu X (2009). Optically controlled locking of the nuclear field via coherent dark-state spectroscopy. Nature.

[60] Press D (2010). Ultrafast optical spin echo in a single quantum dot. Nat. Photonics.

[61] Hu CY, Rarity JG (2011). Loss-resistant state teleportation and entanglement swapping using a quantum-dot spin in an optical microcavity. Phys. Rev. B.

[62] Kawakami E (2014). Electrical control of a long-lived spin qubit in a Si/SiGe quantum dot. Nat. Nanotechnol..

[63] Elzerman JM (2004). Single-shot read-out of an individual electron spin in a quantum dot. Nature.

[64] Kroutvar M (2004). Optically programmable electron spin memory using semiconductor quantum dots. Nature.

[65] Golovach VN, Khaetskii A, Loss D (2004). Phonon-Induced Decay of the Electron Spin in Quantum Dots. Phys. Rev. Lett..

[66] Hu CY, Rarity JG (2015). Extended linear regime of cavity-QED enhanced optical circular birefringence induced by a charged quantum dot. Phys. Rev. B.

[67] Wei HR, Deng FG (2014). Universal quantum gates on electron-spin qubits with quantum dots inside single-side optical microcavities. Opt. Express.

[68] Rosenblum S (2018). A CNOT gate between multiphoton qubits encoded in two cavities. Nat. Commun..

[69] Heo J, Hong C, Choi SG, Hong JP (2019). Scheme for generation of three-photon entangled W state assisted by cross-Kerr nonlinearity and quantum dot. Sci. Rep..

[70] Walls, D. F. & Milburn, G. J. *Quantum Optics* (Springer-Verlag, Berlin, 1994).

[71] Reithmaier JP (2004). Strong coupling in a single quantum dot–semiconductor microcavity system. Nature.

[72] Yoshie T (2004). Vacuum Rabi splitting with a single quantum dot in a photonic crystal nanocavity. Nature.

[73] De Greve K, Press D, McMahon PL, Yamamoto Y (2013). Ultrafast optical control of individual quantum dot spin qubits. Rep. Prog. Phys..

[74] Dory C (2016). Complete Coherent Control of a Quantum Dot Strongly Coupled to a Nanocavity. Sci. Rep..

[75] Fleischhauer M, Imamoglu A, Marangos JP (2005). Electromagnetically induced trans-parency: optics in coherent media. Rev. Mod. Phys..

[76] Arnold C (2012). Optical bistability in a quantum dots/micropillar device with a quality factor exceeding 200000. Appl. Phys. Lett..

[77] Reitzensteina S (2007). AlAs/GaAs micropillar cavities with quality factors exceeding 150.000. Appl. Phys. Lett..

[78] Emary C, Xu XD, Steel DG, Saikin S, Sham LJ (2007). Fast initialization of the spin state of an electron in a quantum dot in the Voigt configuration. Phys. Rev. Lett..

[79] Chen PC, Piermarocchi C, Sham LJ, Gammon D, Steel DG (2004). Theory of quantum optical control of a single spin in a quantum dot. Phys. Rev. B.

[80] Berezovsky J (2006). Nondestructive optical measurements of a single electron spin in a quantum dot. Science.

[81] Berezovsky J, Mikkelsen MH, Stoltz NG, Coldren LA, Awschalom DD (2008). Picosecond Coherent Optical Manipulation of a Single Electron Spin in a Quantum Dot. Science.

[82] Press D, Ladd TD, Zhang B, Yamamoto Y (2008). Complete quantum control of a single quantum dot spin using ultrafast optical pulses. Nature.

